# It's About Time: A Study of Rheumatology Patient Consultation Times

**DOI:** 10.7759/cureus.48007

**Published:** 2023-10-30

**Authors:** Ridda Khan, Salman Khurshid Imami, Saira E Anwer Khan, Shabnam Batool, Faiza Naeem, Muhammad Adeel Zaffar

**Affiliations:** 1 MS Healthcare Management & Innovation, Lahore University of Management Sciences, Lahore, PAK; 2 Partnerships and Programs, Shalamar Medical and Dental College, Lahore, PAK; 3 Rheumatology, Shalamar Hospital/Shalamar Institute of Health Sciences, Lahore, PAK; 4 Sulemand Dawood School of Business, Lahore University of Management Sciences, Lahore, PAK

**Keywords:** quality of care, health education, rheumatology, rheumatological consultation, consultation times

## Abstract

Objective: A study was conducted evaluating the process of a rheumatology consultation.

Methods: Data on consultation times was obtained from 100 patient processes over three months. Prior to data collection, unstructured interviews were conducted with the entire staff of the rheumatology clinic in Shalamar Hospital, to understand the consultation process. Based on this, consultation was divided into distinct segments (vitals, history and examination, specialist registrar consultation, specialist consultation, documentation and exercise/prescription handing over) and data was collected for the time taken for the patient to complete each segment. Designation of the personnel conducting the process, diagnosis, current visit number and general notes were also recorded.

Results: Patients with rheumatoid arthritis (RA) consulted for an average time of 33.4 and 27.4 minutes for new and established patients respectively in our observations. Patients with systemic lupus erythematosus (SLE) on the other hand spent 34.5 and 37 minutes for new and established patients respectively. The greatest time spent during any segment of the consultation was during documentation, which averaged 10 minutes per patient.

Conclusion: Our study found that consultation times at Shalamar Hospital's rheumatology clinic align with international guidelines. Implementing a triaging method could optimize resource allocation, while entrusting specialist nurses with stable patient follow-ups could enhance patient flow and provision of health education.

## Introduction

Consultation time is recognized as a quality indicator by the World Health Organization (WHO) and the International Network of Rational Use of Drugs (INRUD) [1,2]. A systematic review of 67 countries showed that the average time spent during a consultation by 50% of the population of the world is five minutes, though significant disparities exist, ranging from 48 seconds in Bangladesh to 22.5 minutes in Sweden, which may be due to difference in the perceived role of physicians and healthcare providers in each society [2]. It also influences the patient-doctor relationship and quality of care [3,4]. Not only does it reflect upon the satisfaction of the patient but also that of the doctor [4]. The rising population is further burdening the healthcare systems established, leading to primary care physicians (PCP) dissatisfied with the quality of care they provide, reduction in the provision of healthcare services and PCPs feeling rushed at the end of a consultation [2,5].

The consultation times in the developed world are gradually rising [6]. The British Royal College of General Practitioners has recommended that primary care appointments be at least 15 minutes long [7,8]. According to a study in Pakistan by Atif et al. in 2016, more than 10 minutes was considered optimal consultation time [9]. In Egypt more than 30 minutes per patient was considered optimal [10]. The increased time patient spends with the doctor allows for better extraction of information and improved management of multimorbid cases [11]. Notably, Heidari et al. observed greater adherence to medication by the patient and compliance to treatment prescribed by the rheumatologist, underlining the role of thorough patient education, in longer consultations [12].

However, longer consultations do not necessarily translate into better ones according to a Cochrane review of clinical trials [13]. Some evidence on the other hand does suggest that they are better at ascertaining psychological illnesses [14]. They also allow for increased time to be spent on health education and promotion by the physician, allowing for patients' better understanding of their disease, therefore such patients were more likely to be compliant and less reluctant for lifestyle modifications [15].

The current situation in South Asia is not any better than in the rest of the world, where a study on non-communicable diseases (NCDs) done in Pakistan and Bangladesh revealed that wait times could exceed more than five hours while the consultation could be only five minutes [16]. Furthermore, there is a paucity of data on the consultation times of specialists. A survey of Korean rheumatologists by Kim and Kim (2020) showed that the average time spent on consults was 4.8 minutes, far from the perceived optimal time of 17-38 minutes [3].

Currently there is no published study that systematically disassembled the elements of a rheumatological consult, determining the average time spent per segment and analyzing each step within the context of Pakistan. This study was aimed at finding the actual time spent by the patient per segment of a rheumatology consultation for rheumatic diseases.

## Materials and methods

We conducted an observational study on 100 patient processes at the rheumatology outpatient clinic at Shalamar Hospital, Lahore, Pakistan. Institutional review board (IRB) review was waived as this was a study of patient processes, and observations were made from administrative data. Consent for use of administrative data was taken from all patients. Unstructured interviews were conducted with all the healthcare providers prior to the start of the study to understand the processes that were occurring within the clinic to help better define them. The consultation process inside the clinic was divided into segments, and the duration was recorded for each patient at every segment. Data was ensured not to contain any personal identifiers and was collected on the following parameters: diagnosis, time taken by the patient for each segment, type of visit (i.e., new or follow-up), designation of the person attending the patient, referral to any other department, date, day, and time of the visit. It was collected according to the convenience of the investigators at Shalamar Hospital, Lahore from March to May 2023. To reduce potential bias, the healthcare providers were not informed about the data of the specific individuals under observation.

**Figure 1:**
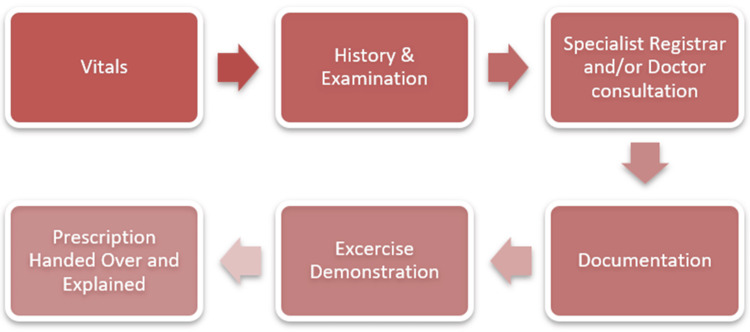
Segments of a rheumatological consultation

The process inside the clinic can be divided into the following steps as seen in Figure 1:

The segments of the consultation were divided into: Vitals (weight, blood pressure, heart rate and temperature), which are noted by the nurse for every patient; History and Examination, which involves review of presenting complaints, symptoms, diagnostic reports, and physical examination and is carried out by the Post-Graduate Resident (PGR) or Medical Officer (MO); Specialist Registrar Consultation, carried out after History and Examination, which is a preliminary consult for all patients; Specialist Rheumatology Consultation, which is carried out for patients who have requested themselves or for complications that require an expert opinion, where the person who has taken the history of the patient (MO or the PGR) accompanies the patient for consultation; Documentation, which is carried out by a designated operator who types all the notes from the token slip which includes vitals, diagnosis, treatment plan, symptoms, and the dosage of the drug therapy; and Exercise demonstration and Prescription handing over, which are both carried out by the specialist nurse. Exercises are demonstrated and the prescription is explained in layman's terms.

The data collected was analysed using descriptive statistics only in terms of mean times spent in each segment of the consultation. 

Inclusion criteria

Data of individuals age 18 years and above, attending the rheumatology outpatient department (OPD), with definite diagnosis of rheumatic diseases was included. Inclusion was restricted to the timeframe of March to May 2023. Both newly diagnosed patients (those receiving their diagnosis or having their first confirmed visit) and established patients (those on follow-up appointments) were encompassed within the study.

Exclusion criteria

Any of the above criteria not met.

## Results

Of 100 patients 98 fit our inclusion criteria and were included in our study, two patients excluded were improper referrals with no current or previous history of rheumatic diseases.

The average time spent during each segment of the consultation was two minutes for Vitals, eight minutes for History and Examination, three minutes for Specialist Registrar Consultation, four minutes for Rheumatologist Consultation, 10 minutes for Documentation and one minute for Exercise Demonstration and Prescription Handing Over as described in Table 1.

**Table 1:** Average time spent by all patients during each segment of the consultation SD; Standard Deviation

Segment of Consultation	Average Time in Minutes Spent Per Patient (Mean±SD)
Vitals	2±1.0
History and Examination	8±5.5
Specialist Registrar Consult	3±4.4
Rheumatologist Consult	4±4.9
Documentation	10±6.4
Exercise and Prescription Handing Over	1±1.5

**Figure 2:**
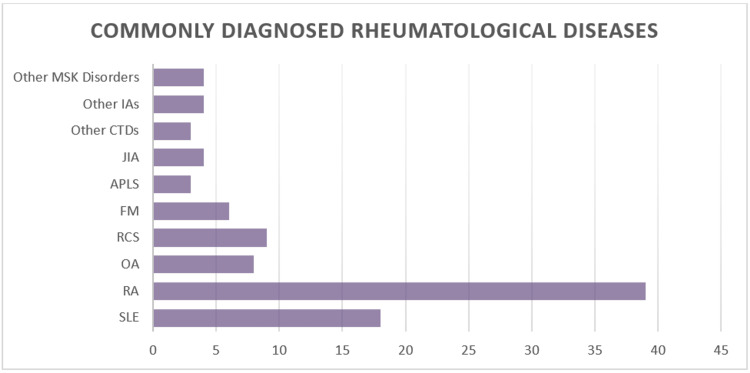
Commonly diagnosed rheumatological diseases SLE; Systemic Lupus Erythematosus, RA; Rheumatoid Arthritis, OA; Osteoarthritis, RCS; Rotator-Cuff Syndrome, FM; Fibromyalgia, APLS; Anti-phospholipid Syndrome, JIA; Juvenile Idiopathic Arthritis, Other CTDs (Connective Tissue Diseases): Systemic Sclerosis and Mixed Connective Tissue Disease; Other IAs (Inflammatory Arthropathies): Inflammatory Arthritis and Ankylosing Spondylitis, Other MSK (Musculoskeletal) disorders: Back Pain, Spasm and Cramps

Thirty-nine patients were diagnosed with rheumatoid arthritis (RA), of which nine were newly diagnosed, making it the most common diagnosis amongst the patients included as seen in Figure 2. The average consultation for RA lasted 33.4 and 27.4 minutes for new and established patients respectively. Systemic lupus erythematosus (SLE) was the second most common disease with a total of 18 patients presenting to the clinic. Of these, only two were new patients and the rest were established. The average time observed for the whole consultation period for these patients was 34.5 minutes and 37 minutes for new and established patients. The data for consultation times for RA and SLE patients in each segment is given in Table 2. There were nine patients diagnosed with rotator-cuff syndromes, which was the next most diagnosed condition that overlapped with RA in three patients. These patients spent on average 28 minutes per consultation.

**Table 2:** Average time spent by RA and SLE patients as a new or established patient during each segment of consultation SLE; Systemic Lupus Erythematosus, RA; Rheumatoid Arthritis, SD; Standard Deviation

Disease	SLE (n=18)			RA (n=39)		
Visit Type	Total Time (Mean±SD)	New Patient (Mean±SD)	Follow-Up Patient (Mean±SD)	Total Time (Mean±SD)	New Patient (Mean±SD)	Follow-Up Patient (Mean±SD)
Vitals	3	3	3	2	2	2
History and Examination	10±6.7	16.5±13.4	10±5.8	9±5.9	8.8±2.9	9±6.5
Specialist Registrar Consultation	4±6.2	0	5±6.4	3±3.8	2.5±2.1	3±4.1
Rheumatologist Consultation	8±5.7	4.5±6.4	8±5.7	4±3.0	5.4±2.8	4±3.1
Documentation (Prescription Preparation)	11±6.3	10.5±6.4	11±6.5	11±6.5	14.7±9.6	10±5.4
Exercise	-	-	-	1±1.1	1.5±1.5	1±1.0

During our analysis of various segments of the consultation we discovered the patients with SLE spent most time overall during the consultation. On average during history and examination (10 minutes), specialist registrar consultation (four minutes), rheumatologist consultation (eight minutes) and prescription preparation (11 minutes) by all SLE patients as seen in the data given in Table 2. Average time when stratified for new and established patients further showed that only the follow-up cases were dealt with by the registrar with an average of five minutes spent per patient. The maximum time spent by the rheumatologist was 24 minutes during this segment with a new, multimorbid patient with seronegative RA, rotator-cuff syndrome, and osteoporosis.

## Discussion

Our study dissected the events during a rheumatological consultation. The average time during a complete consultation at the Rheumatology outpatient clinic in Shalamar Hospital came out to be 28 minutes. According to the study by Deveugele (2002), consultation time varied from country to country, amongst the six European countries surveyed [17]. It was seen to be dependent on the volume of patients, nature of complaints (new vs chronic), psychosocial issues, and age of the patients. The British Society of Rheumatology further recommends that a new RA patient spends at least 30 minutes during the first appointment and the established patient spends 15 minutes during follow-up consultations [18]. The recommendations however have not divided the process of consultation into segments like in the above study. As per the mentioned guidelines, the rheumatology department allocates sufficient time for both initial (34.9 minutes) and follow-up patient (29 minutes) visits, enabling the delivery of high-quality care.

A thorough literature review revealed a Korean study by Kim and Kim, 2020, previously mentioned in the Introduction, focusing on rheumatologists’ perspective of consultation times, which divided the process of consultation into segments that were comparable with our study. Furthermore, the study also determined the perceived optimal time for consultation in patients with RA and SLE. Our study indicated that the average time allocated to new patients of RA was 34.9 minutes, and for established patients, it was 29 minutes. This is in line with their perceived optimal time of 34.8 minutes for new patients and 17.6 minutes for established patients Table 3 [3].

**Table 3:** Comparison of segments of the rheumatology consultation of RA patients with the optimal time derived in the Korean paper RA; Rheumatoid Arthritis, SD; Standard Deviation

	RA new patient			RA established patient		
Segment of Consultation	Perceived optimal time [3] (Mean±SD)	Observed actual time in minutes in Rheumatology Clinic, Shalamar Hospital (Mean±SD)	Variance	Perceived optimal time [3] (Mean±SD)	Observed actual time in minutes in Rheumatology Clinic, Shalamar Hospital (Mean±SD)	Variance
Vitals	7.9±10.4	2.0	-5.9	2.9±2.0	2.0	-0.9
History & Examination	12.4±7.7	8.8±2.9	-3.6	6.2±4.3	9.0±6.5	+2.8
Registrar and Specialist Consultation & Exercise Demonstration	10±6.7	9.4±6.4	-0.6	5.9±3.6	8.0±8.2	+2.1
Documentation	4.6±2.8	14.7±9.6	+10.1	2.6±2.0	10.0±5.4	+7.4

Likewise, in the case of SLE patients, the average consultation times were 34.5 minutes for new patients and 37 minutes for established patients. Comparatively, the Korean study identified optimal durations of 38.8 minutes for new patients and 21.6 minutes for established patients [3].

The time spent during first visits by both RA and SLE patients was less than the optimal time described in the literature and during follow-up visits patients spent on average greater times. Interviews with the rheumatologist disclosed that this time discrepancy during the follow-up visits can be a local dynamic in the Pakistani setup. First visits are usually short in our setting because the rheumatology clinic is their first encounter with healthcare, and the rheumatologist functionally acts as a general physician, prescribing adequate investigations and starting empirical treatment, while the task of specialized disease management and health education is deferred until the subsequent appointment leading to the deviation from the norm. This contrasts with the guidelines followed by the National Health Service (NHS), where it is recommended that first-line investigations are carried out by general physicians before referral preventing any delays in management of the patient [19]. Unfortunately, in our setup this must wait until the patient returns for follow-up, as our referral system is in its preliminary stages and requires rigorous overhauling [20]. Also, during the subsequent visits the established patient’s knowledge about the disease, specifically RA, was seen to increase in the study Bech et al. (2020), which increases the possibility for a detailed discussion regarding the illness during these visits thus taking up more consultation times [21].

We also discovered that most of the time was taken up by a non-value adding activity of documentation which averaged to 10 minutes per patient. This adds to increased wait times at the clinic. Notably, it was seen that the specialist registrar triaged the patients, escalating care to the rheumatologist when needed. Moreover, most follow-up cases were only managed by the registrar themselves, improving efficiency, increasing patient turnover, and resulting in better resource allocation. A randomized controlled trial by Primdahl et al. was conducted in 2013 on patients with RA aimed to uncover potential disparities in outcomes between patients monitored by nurses or physicians [22]. The study findings indicated that there was no notable variation in disease activity indices among patients attended to by either group. This shows that management of stable follow-up patients with low disease activity can further be delegated to specialist nurses in our setup freeing up valuable time for detailed consultations with patients who are non-compliant or adherent to treatment.

Furthermore, an educational component is also mentioned during the process of consultation in the Korean study, where the average perceived optimal time ranges from three to eight minutes. This in comparison to the time (1±1.5 minutes) the specialist nurse spends during exercise demonstration, and prescription handing over, with the patient in our setup shows a difference in practices between the two setups. It is also seen that longer consultation and education lead to reduced polypharmacy and reduction in overutilization of healthcare resources [3].

The psychosocial aspect of chronic rheumatic diseases cannot be undermined. Understanding the psychological and social needs of the patients takes time and effort therefore longer consultations as seen in our study. Although a Cochrane review of five studies suggests that longer consultation times do not necessarily correlate with better care, recent evidence does show that it's better for dealing with patients with psychosocial issues [13,14].

Since our study was done at a trust hospital with subsidized rates for consultations making it affordable for a larger subset of patients, increasing inflow, possibly skewing our observations resulting in reduced time spent per patient. This observation can be compared to an Iranian study by Heidari et al. in 2019. However, neither of the studies defined optimal consultation times and the Iranian study did not quantify any data regarding consultation times, it being a qualitative study [12].

Strengths and limitations

The strengths of our study lie in the fact that it is a first-of-its-kind study to be done in the scope of Pakistan and especially in the field of rheumatology. Its strength lies in the fact that the data was collected in a real-world setup, without any interference by the investigators. Furthermore, the staff at the clinic, including the doctors, nurses, and paramedics, were partially blinded. They were not made aware of the patients of whom the data was collected. This was done to minimize the Hawthorne effect.

Since our study was an observational analysis, data was collected in a non-randomized manner according to convenience, therefore our data may be biased. We conducted our study in a rheumatology clinic of a trust, tertiary care hospital, which may limit the generalizability of our study as all the patient population was not studied equally, and patient demographics were not included. Lastly, it's important to note that the data collection period was relatively brief. Furthermore, no tests of statistical significance were used to analyse the data making this study purely a survey of data. 

To ensure a more comprehensive understanding, conducting additional randomized controlled trials, from insights gained from this study, over an extended timeframe with standardized patients would be valuable. These future trials could further delve into the assessments of both patients and rheumatologists regarding their perceptions of consultation times considered adequate. 

## Conclusions

Our results showed that consultation times in the rheumatology clinic at Shalamar Hospital were comparable to international guidelines. The consultation times for both new and established patients with RA and SLE were adequate. The established patients had a greater-than-average consultation time when compared to new patients. The most time-consuming step of the consultation was documentation, which needs to be streamlined and made efficient. Furthermore, there is a need to introduce a triaging method that can effectively allocate existing resources and enhance the provision of specialized patient care. Follow-ups of stable patients can be delegated to specialist nurses. This will allow for increased patient inflow, faster turnover, and improved provision of health education.

## References

[1] Wang Q, Adhikari SP, Wu Y (2022). Consultation length, process quality and diagnosis quality of primary care in rural China: a cross-sectional standardized patient study. Patient Educ Couns.

[2] Irving G, Neves AL, Dambha-Miller H, Oishi A, Tagashira H, Verho A, Holden J (2017). International variations in primary care physician consultation time: a systematic review of 67 countries. BMJ Open.

[3] Kim HA, Kim MG (2020). A survey study on rheumatologist consultation time in Korean hospitals. J Rheum Dis.

[4] Elmore N, Burt J, Abel G, Maratos FA, Montague J, Campbell J, Roland M (2016). Investigating the relationship between consultation length and patient experience: a cross-sectional study in primary care. Br J Gen Pract.

[5] Sampson R, O'Rourke J, Hendry R, Heaney D, Holden S, Thain A, MacVicar R (2013). Sharing control of appointment length with patients in general practice: a qualitative study. Br J Gen Pract.

[6] Orton PK, Pereira Gray D (2016). Factors influencing consultation length in general/family practice. Fam Pract.

[7] Oxtoby K (2010). Consultation times. BMJ.

[8] Gong X, Hou M, Guo R, Feng XL (2022). Investigating the relationship between consultation length and quality of tele-dermatology E-consults in China: a cross-sectional standardized patient study. BMC Health Serv Res.

[9] Atif M, Sarwar MR, Azeem M, Naz M, Amir S, Nazir K (2016). Assessment of core drug use indicators using WHO/INRUD methodology at primary healthcare centers in Bahawalpur, Pakistan. BMC Health Serv Res.

[10] Elkahky AA, Salem AM (2014). WHO/INRUD drug use indicators at primary healthcare centers in Alexandria, Egypt. J Taibah Univ Med Sci.

[11] Howie JG, Porter AM, Forbes JF (1989). Quality and the use of time in general practice: widening the discussion. BMJ.

[12] Heidari P, Cross W, Weller C, Team V, Nazarinia M, Crawford K (2019). Rheumatologists' insight into medication adherence in patients with rheumatoid arthritis: a qualitative study. Int J Rheum Dis.

[13] Wilson AD, Childs S (2006). Effects of interventions aimed at changing the length of primary care physicians' consultation. Cochrane Database Syst Rev.

[14] Hutton C, Gunn J (2007). Do longer consultations improve the management of psychological problems in general practice? A systematic literature review. BMC Health Serv Res.

[15] Wilson A, Childs S (2002). The relationship between consultation length, process and outcomes in general practice: a systematic review. Br J Gen Pract.

[16] Wright J, Mazumdar P, Barua D (2020). Integrating depression care within NCD provision in Bangladesh and Pakistan: a qualitative study. Int J Ment Health Syst.

[17] Deveugele M, Derese A, van den Brink-Muinen A, Bensing J, De Maeseneer J (2002). Consultation length in general practice: cross sectional study in six European countries. BMJ.

[18] Kennedy T, McCabe C, Struthers G (2005). BSR guidelines on standards of care for persons with rheumatoid arthritis. Rheumatology (Oxford).

[19] (2023). Rheumatoid arthritis in adults: management. https://www.nice.org.uk/guidance/ng100/chapter/Recommendations.

[20] Siddiqi S, Kielmann A, Khan M, Ali N, Ghaffar A, Sheikh U, Mumtaz Z (2001). The effectiveness of patient referral in Pakistan. Health Policy Plan.

[21] Bech B, Lykkegaard JJ, Lundbak T (2020). Patient-Initiated Follow-Up (PIFU) as reorganized support for increased patient involvement - focus group discussions among patients' with inflammatory arthritis. BMC Rheumatol.

[22] Primdahl J, Sørensen J, Horn HC, Petersen R, Hørslev-Petersen K (2014). Shared care or nursing consultations as an alternative to rheumatologist follow-up for rheumatoid arthritis outpatients with low disease activity--patient outcomes from a 2-year, randomised controlled trial. Ann Rheum Dis.

